# Context aware deep learning for brain tumor segmentation, subtype classification, and survival prediction using radiology images

**DOI:** 10.1038/s41598-020-74419-9

**Published:** 2020-11-12

**Authors:** Linmin Pei, Lasitha Vidyaratne, Md Monibor Rahman, Khan M. Iftekharuddin

**Affiliations:** Vision Lab, Electrical and Computer Engineering, Old Dominion University, Norfolk, VA 23529 USA

**Keywords:** CNS cancer, Information technology, Diagnostic markers, Predictive markers, Biomedical engineering

## Abstract

A brain tumor is an uncontrolled growth of cancerous cells in the brain. Accurate segmentation and classification of tumors are critical for subsequent prognosis and treatment planning. This work proposes context aware deep learning for brain tumor segmentation, subtype classification, and overall survival prediction using structural multimodal magnetic resonance images (mMRI). We first propose a 3D context aware deep learning, that considers uncertainty of tumor location in the radiology mMRI image sub-regions, to obtain tumor segmentation. We then apply a regular 3D convolutional neural network (CNN) on the tumor segments to achieve tumor subtype classification. Finally, we perform survival prediction using a hybrid method of deep learning and machine learning. To evaluate the performance, we apply the proposed methods to the Multimodal Brain Tumor Segmentation Challenge 2019 (BraTS 2019) dataset for tumor segmentation and overall survival prediction, and to the dataset of the Computational Precision Medicine Radiology-Pathology (CPM-RadPath) Challenge on Brain Tumor Classification 2019 for tumor classification. We also perform an extensive performance evaluation based on popular evaluation metrics, such as Dice score coefficient, Hausdorff distance at percentile 95 (HD95), classification accuracy, and mean square error. The results suggest that the proposed method offers robust tumor segmentation and survival prediction, respectively. Furthermore, the tumor classification results in this work is ranked at second place in the testing phase of the 2019 CPM-RadPath global challenge.

## Introduction

Gliomas are the most common primary brain malignancies, with varying degrees of aggressiveness, variable prognosis and various heterogeneous regions^1^. In the US, the overall average annual age-adjusted incidence rate for all primary brain and other central nervous system (CNS) tumors has been reported as 23.03 per 100,000 population during 2011–2015^2^. For patients with malignant tumors, the estimated 5- and 10-year relative survival rates are 35.0% and 29.3%, respectively, according to a report from 2011–2015^2^. The median survival period of patients with glioblastoma (GBM) is about 12–15 months^3^. Diagnosis of tumor subtype and grade is vital for treatment planning and prognosis of the patients. According to a 2016 report of World Health Organization (WHO), classification of tumors in the CNS is based on both phenotype and genotype (i.e., *IDH* mutation and *1p/19q codeletion* status)^4^. However, structural imaging such as magnetic resonance imaging (MRI) is continued to be used for identifying, locating, and classifying brain tumors^5–8^. Tumor subtypes include diffuse astrocytoma, *IDH*-wild/-mutant type, oligodendroglioma, *IDH*-mutant and *1p/19q*-codeleted, glioblastoma, *IDH*-wildtype, etc.^4^. Traditional machine learning-based methods, such as support vector machines (SVM), k-nearest neighbors algorithm (KNN), and random forest (RF) are generally utilized for brain tumor analysis^9–15^. However, these methods have the common limitation of hand-crafted feature extraction in the modeling phase.

Deep learning-based methods overcome the drawback of hand-crafted feature extraction. Deep learning has made it possible to build large-scale trainable models that have the capacity to learn the optimal features required for a given task. Deep learning is powerful and outperforms traditional machine learning in many fields, such as computer vision^16–18^, medical image segmentation^19,20^, and speech recognition^21^. Deep learning is fundamentally composed of a deep neural network structure with several layers. An artificial neural network utilizes a backpropagation algorithm to decrease the error between the prediction and true value. However, training artificial neural network models becomes more difficult as the number of layers increase^22^. Deep neural network training has been feasible since the mid-2000s, which brought about increased availability of large datasets and hardware improvements.

**Figure 1:**
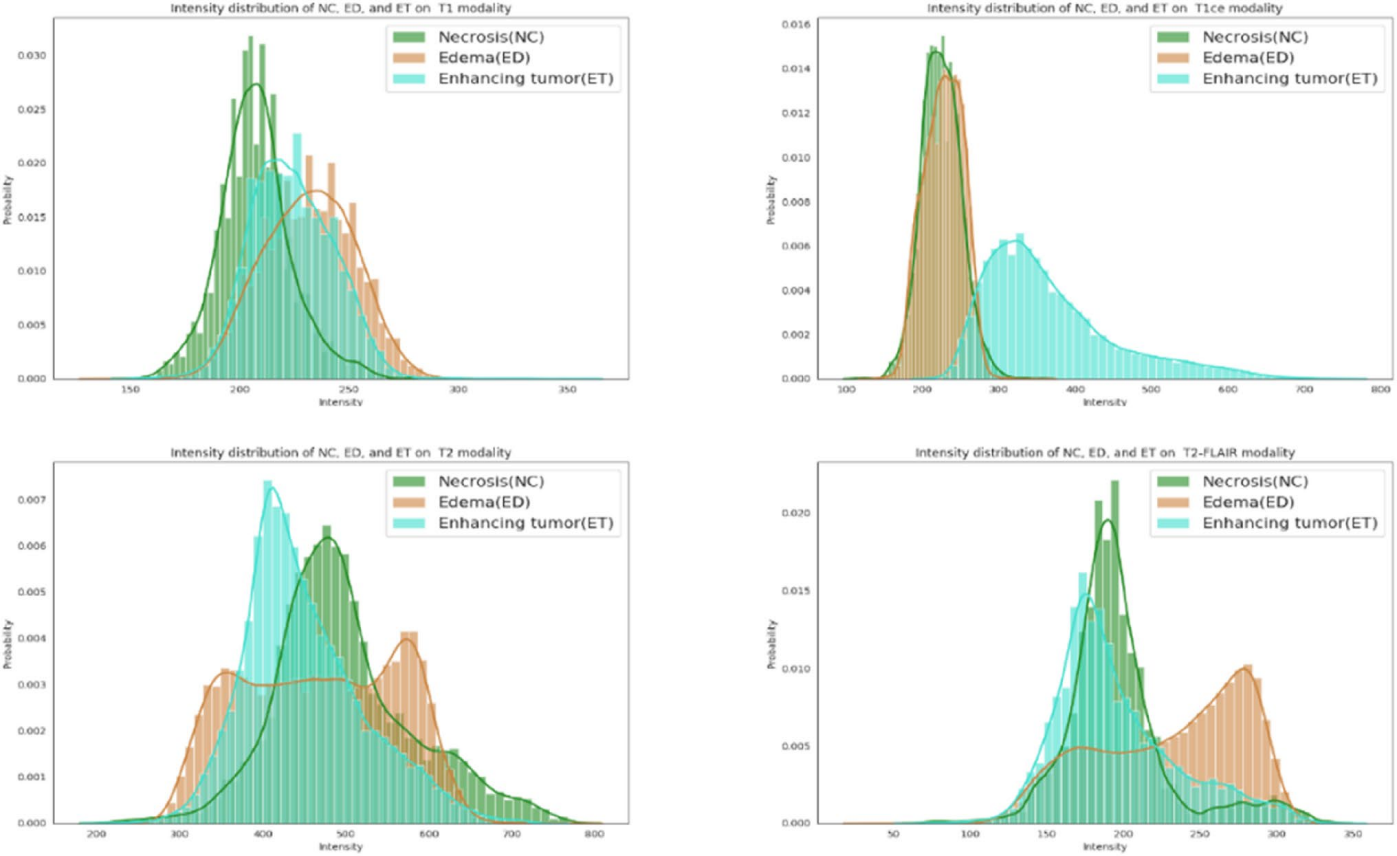
Intensity distribution of necrosis, edema, and enhancing tumor on T1 (top left), T1ce (top right), T2 (bottom left), and FLAIR (bottom right) images of one case.

As a standard protocol for brain tumor characterization, MRI is able to capture a diverse spectrum of tumor phenotypes^23^. Multimodal MRI (mMRI) provides comprehensive tumor information. For example, post-contrast T1-weighted (T1ce) images are well-known to be correlated with blood brain barrier (BBB) disruption, while T2-weighted (T2) and T2 Fluid Attenuated Inversion Recovery (FLAIR) images are well-known for capturing tumor margins and peritumoral edema^23^. This suggests that the phenotypic differences at the cellular level are also reflected in the imaging phenotype (appearance and shape). While mMRI captures comprehensive brain tumor information, extracting this information through brain tumor analysis, such as tumor segmentation, remains challenging because of the similar phenotypic appearance of abnormal tissues in mMRI images. Figure 1 shows the intensity distribution of three types of abnormal brain tissues in T1, T1ce, T2, and FLAIR images for a representative case. These intensity distributions are highly similar for tumor tissues for all patients in this study. While on T1ce image, enhancing tumor (ET) is easily separable from others, the necrosis (NC) and peritumoral edema (ED) have nearly the same intensity distribution.

**Figure 2:**
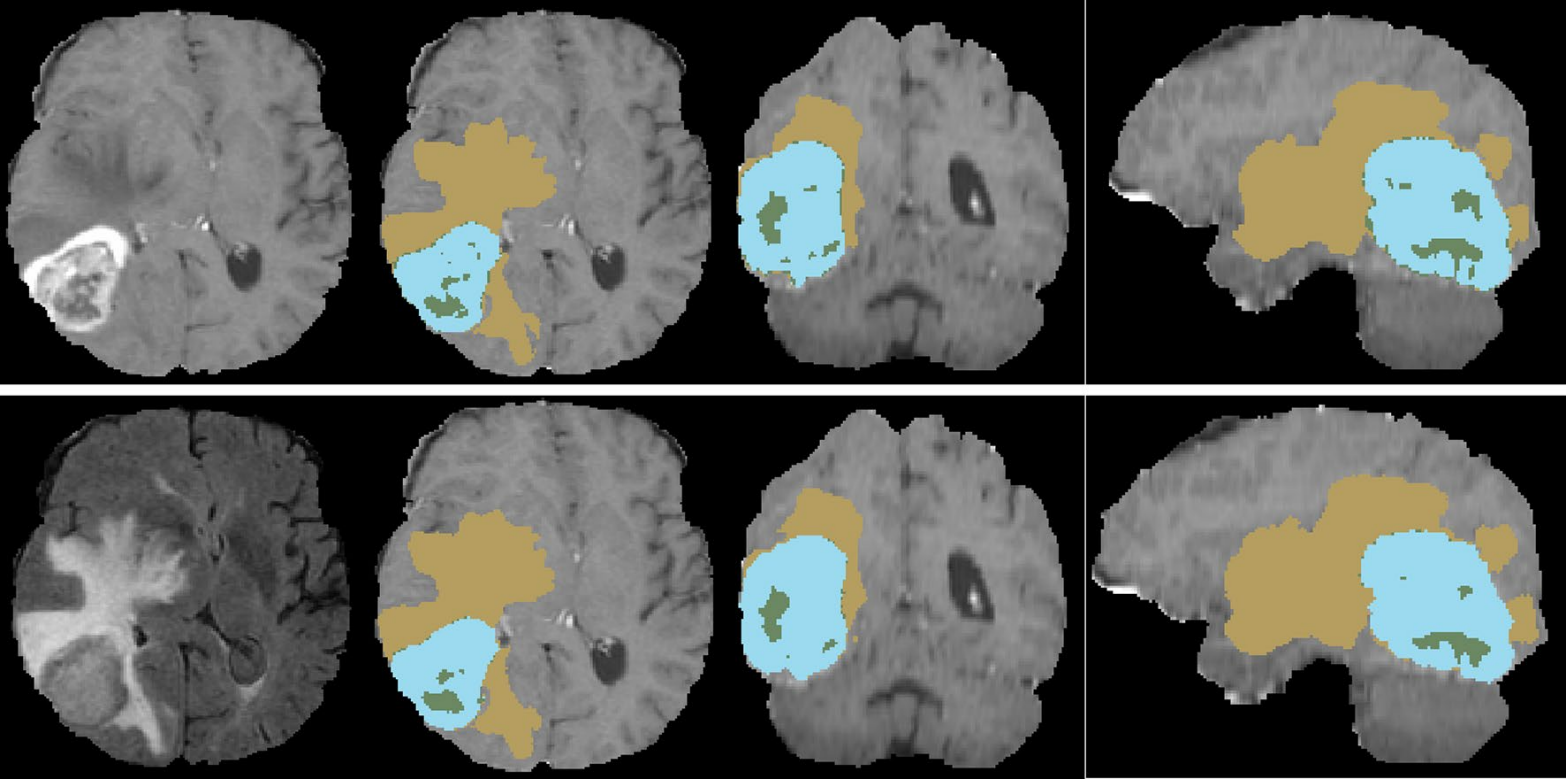
Comparison of tumor segmentation using the proposed method and ground truth. Top row from left to right: T1ce image, segmented tumor overlaid with T1ce in axial view, in coronal view, and in sagittal view. Bottom row from left to right: FLAIR image, ground truth overlaid with T1ce in axial view, in coronal view, and in sagittal view.

Brain tumors have been studied for many years. However, most works study tumor segmentation, classification, and overall survival prediction independently, ignoring the underlying relationship among these critical analysis tasks. In this work, we propose a complete framework for brain tumor study, including tumor segmentation, subtype classification, and overall survival prediction by analyzing mMRI via a deep learning-based neural network architecture.

## Results

**Figure 3:**
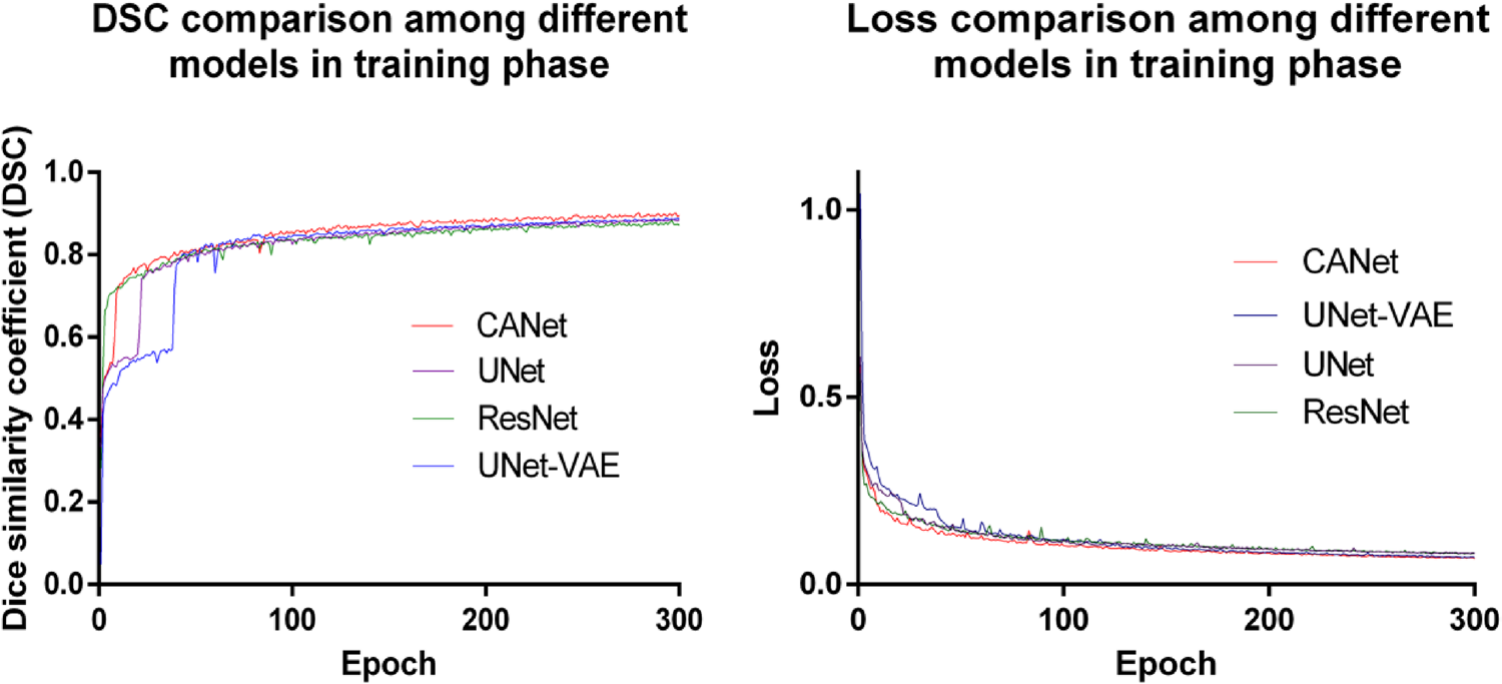
The DSC (left) and loss (right) comparison with other models in training phase.

Experiment 1: Brain tumor segmentation. Figure 2 shows a visual comparison of tumor tissue segmentation in axial, coronal, and sagittal views for a representative case for BraTS 2019. The Dice similarity coefficient (DSC) and training loss changes are shown in Fig. 3. We stop training CANet at epoch 300 as we observe that further improvements in DSC and training loss are not significant with respect to the hefty training time associated with more epochs. The quantification performance of the validation dataset offered by online evaluation is shown in Table 1. For a performance comparison, we also apply three popular architectures, such as ResNet^24^, UNet^19^, and UNet-VAE^25^ to the BraTS 2019 validation dataset (125 cases), and summarize results in Table 1. Overall, Table 1 shows that the proposed CANet achieves significantly better validation results compared to the generic architectures in literature. Therefore, we pick the CANet as the best performing model to proceed to the testing phase.

**Figure 4:**
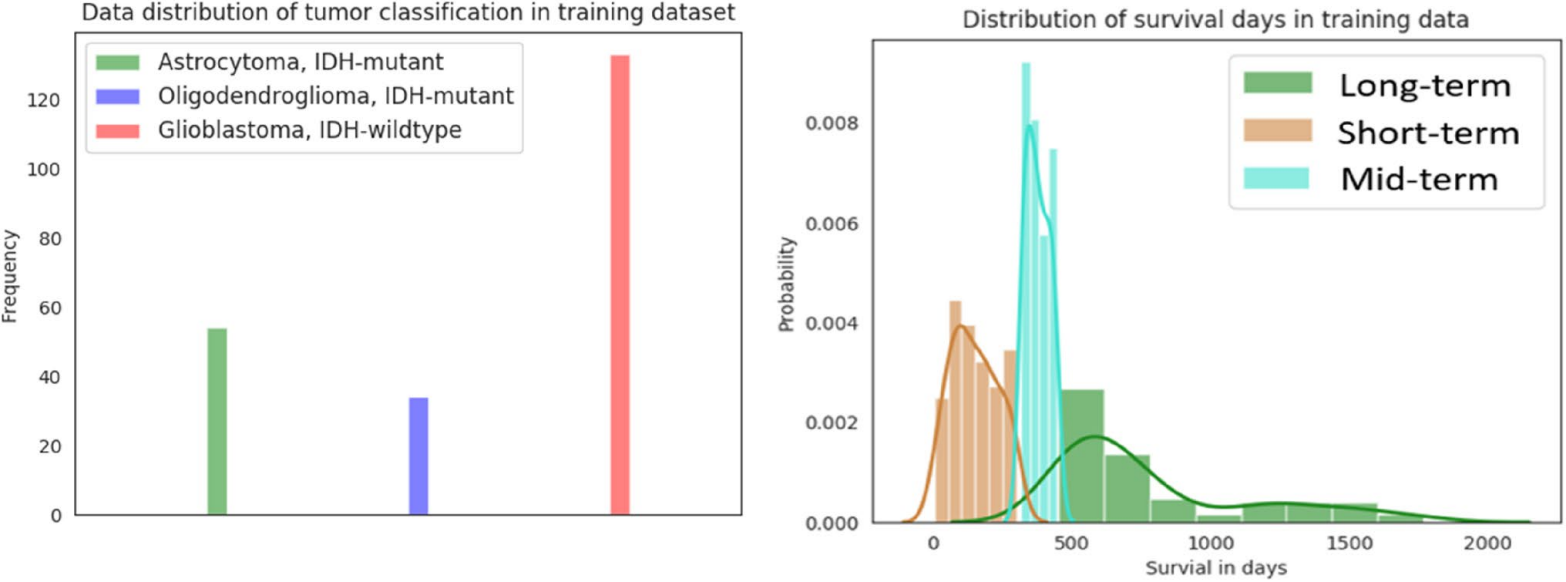
Data distribution of training data for tumor classification and overall survival prediction. (Left) Frequency counts of cases for different classes of tumor. (Right) Distribution of survival days for short-term (< 10 months), mid-term (between 10–15 months), and long-term (> 15 months) categories.

**Table 1:** Tumor segmentation performance comparison with other popular architectures in the validation and testing datasets.

Architecture	Phase	Cases	Dice_ET	Dice_WT	Dice_TC	HD95_ET	HD95_WT	HD95_TC
ResNet	Valid	125	0.732	0.898	0.802	5.213	6.558	8.007
UNet	Valid	125	0.747	0.893	0.808	4.143	5.167	7.502
UNet-VAE	Valid	125	0.769	0.898	0.800	3.554	6.575	7.859
CANet	Valid	125	0.773	0.905	0.815	3.220	4.916	6.809
CANet	Test	252	0.821	0.895	0.835	3.319	4.897	6.712

**Table 2:** Online evaluation of tumor classification on CPM-RadPath 2019 validation and testing datasets.

Phase	Case	Dice	Average	Kappa	Balance_acc	F1_micro
Validation	35	0.749	0.764	0.715	0.749	0.829
Test	142	0.639	NA	0.442	0.639	0.657

**Table 3:** Survival prediction performance of the validation dataset obtained from online evaluation.

Phase	Accuracy	MSE	medianSE	stdSE	SpearmanR
Validation	0.586	79,146	24,362	113,801	0.502
Testing	0.484	334,492	36,923	809,883	0.276

**Table 4:** Performance comparison of overall survival prediction using different methods on BraTS 2019 validation dataset.

Method	Accuracy	MSE	medianSE	stdSE	SpearmanR
Machine learning	0.483	128,594	20,898	233,919	0.044
The proposed method	0.586	79,146	24,362	113,801	0.502

**Table 5:** Age and survival days comparison based on gender.

	HGG					LGG					Overall				
	Case	AA	MA	AS	MS	Case	AA	MA	AS	MS	Case	AA	MA	AS	MS
Male	58	60.9	60.6	520.6	426.5	7	51.7	53.8	1188.6	788	65	59.9	59.3	592.6	448
Female	31	59.3	61.5	433.1	291	10	48.1	50.1	1207.7	983	41	56.6	57.6	622	370

**Table 6:** P-value using ANOVA.

	$$p-\mathrm{value}$$
Gender	0.1636
Age	0.101

The proposed method is tested using a dataset of 252 cases sources from BraTS 2019, BraTS 2020, and TCIA datasets as discussed in the data description section. The testing data evaluation offers average DSC of 0.821, 0.895, and 0.835 for ET, WT, and TC, respectively. We also compare CANet performance between validation and testing data in Table 1. Accordingly, we observe that the DSC of WT is 1% lower in testing phase compared to validation. However, DSC of ET and TC shows 5% and 2% improvement in the testing phase. In addition, we also compute the Hausdorff distance which measures the metric space between the segmentation and ground truth^26^. A smaller Hausdorff distance implies a greater similarity between two images. Accordingly, the average Hausdorff distance at 95th percentile (HD95) in the testing phase is 3.319 mm for ET, 4.897 mm for WT, and 6.712 mm for TC, respectively. We notice that the Hausdorff distance measures in testing phase are constantly lower than that of the validation phase.

As the comparison in Table 1 shows, the proposed CANet offers slight improvements in Dice coefficient measures over other methods. Specifically, CANet achieves a 1–4% improvement in ET, 1% in WT, and 1% in TC segmentation improvement comparing to others. More prominently, CANet achieves significant improvements in the HD95 measure, with a 0.3–2 mm reduction for ET, 0.2–1.6 mm reduction for WT, and 0.7–1.2 mm reduction for TC, respectively. Additionally, the CANet architecture is designed to learn several tasks beyond just tumor segmentation, such as tumor subtype classification, and patient survival prediction, respectively.

Experiment 2: Tumor classification. We apply the proposed method to CPM-RadPath 2019 validation dataset, then wrap the trained model using Docker^27^, and share with the CPM-RadPath Challenge organizer. In the testing phase, the organizer executes the wrapped algorithm to obtain tumor subtype classification result for the final competition. The performance of validation and testing datasets are shown in Table 2. In the testing phase, our result is ranked at second place^28^.

**Table 7:** Gender information in the experimental training data summary.

	Gender			Total
	Male	Female	Unknown	
Tumor segmentation	90	76	169	335
Tumor classification	52	46	121	221
Survival prediction	65	41	104	210

Experiment 3: Overall survival prediction. BraTS 2019 offers a validation dataset with 29 cases for online evaluation. We achieve a validation accuracy of 0.586 as shown in Table 3. In the testing phase the proposed method obtains an accuracy of 0.484 with mean square error (MSE) of 334,492 with a total of 124 testing cases.

### Novel contribution

To the best of our knowledge, brain tumor segmentation, tumor subtype classification, and overall survival prediction have been studied independently, ignoring the inherent relationship among them. In this work, we propose an integrated method for brain tumor segmentation, tumor subtype classification, and overall survival prediction using deep learning and machine learning methods. The specific contributions are as follows.

First, we propose a context aware deep learning-based method for brain tumor segmentation. Second, we utilize a hybrid method for overall survival predication. Specifically, we extract high-dimensional features using the proposed context encoding based convolutional neural network (CANet), and subsequently perform a traditional machine learning method to select features, and finally apply a linear regression method for overall survival prediction. Third, in the framework, all sub-tasks are intercorrelated via the proposed deep learning methods, rather than studied independently.

Finally, though new WHO tumor classification criteria indicate the use of both pathology images and molecular information along with MRI, the proposed method is effective in tumor classification using structural MRI data only. The proposed tumor classification results in this work is ranked at second place in the testing phase of the 2019 CPM-RadPath global challenge among 86 registered teams.

## Conclusion and future work

In this study, we investigate multiple tasks in brain tumor analysis by applying deep learning-based methods to structural multimodal MRI (mMRI) images. These brain tumor analysis tasks consist of tumor segmentation, tumor classification, and overall survival prediction. We propose a context aware deep learning method for tumor segmentation since the context encoding module captures global context encoding features. The segmented tumor is then used for tumor classification by utilizing a 3D CNN. Moreover, we also propose a hybrid method for overall survival prediction. Specifically, we obtain high-dimensional feature extraction using front-end of the CANet, then apply the least absolute shrinkage and selection operator (LASSO) feature selection method to these extracted features, and finally implement an overall survival prediction method based on the selected features.

Note that the performance of complex deep-learning methods developed solely for a challenge such as BraTS 2019 may be compromised due to small sample size, data imbalance, and image quality. However, we have addressed these possible issues in this study by incorporating substantial amounts of additional data for each task from several public datasets. These additional samples are exclusively utilized to enhance the testing of the proposed methods for robustness and generalizability. To further mitigate such problems and obtain generalized training, we implement a subregion-based image analysis scheme, and data augmentation methods that virtually increases the training sample size as discussed in a later section. Consequently, the results demonstrate that the proposed methods show state-of-the-art performance in all three tasks with sufficient robustness to handle data from multiple datasets. In future, we plan extensions to the proposed architecture by integrating whole slide image and molecular genetic features for tumor classification following new WHO criterion^4^.

## Discussion

Deep learning-based methods have been widely applied to many fields and have achieved state-of-the-art performance. However, brain tumor segmentation poses several unique challenges. First, image quality has a critical impact on segmentation performance. For example, blurred images result in poor outcomes. Second, image pre-processing steps also have an impact on the performance. For example, intensity normalization across cases is critical for tumor segmentation. Third, tumor tissue heterogeneity may pose a serious challenge to the developing an effective method. Finally, data imbalance is common and poses another intricate challenge for the use of deep learning. Figure 4 shows the data distribution in the training phase for tumor classification and overall survival prediction in our experiments. Cases of glioblastoma make up more than 50% of the training data. In survival prediction, range of survival days for mid-term survival is too narrow compared to the short- and long-term ranges, creating a data imbalance. This data imbalance can result in misclassification. In segmentation step, samples for edema is generally much more than other abnormal tissues. In order to address the potential data imbalance problem in tumor segmentation, we implement tumor segmentation based on MRI sub-regions, rather than using each abnormal tissue individually.

**Figure 5:**
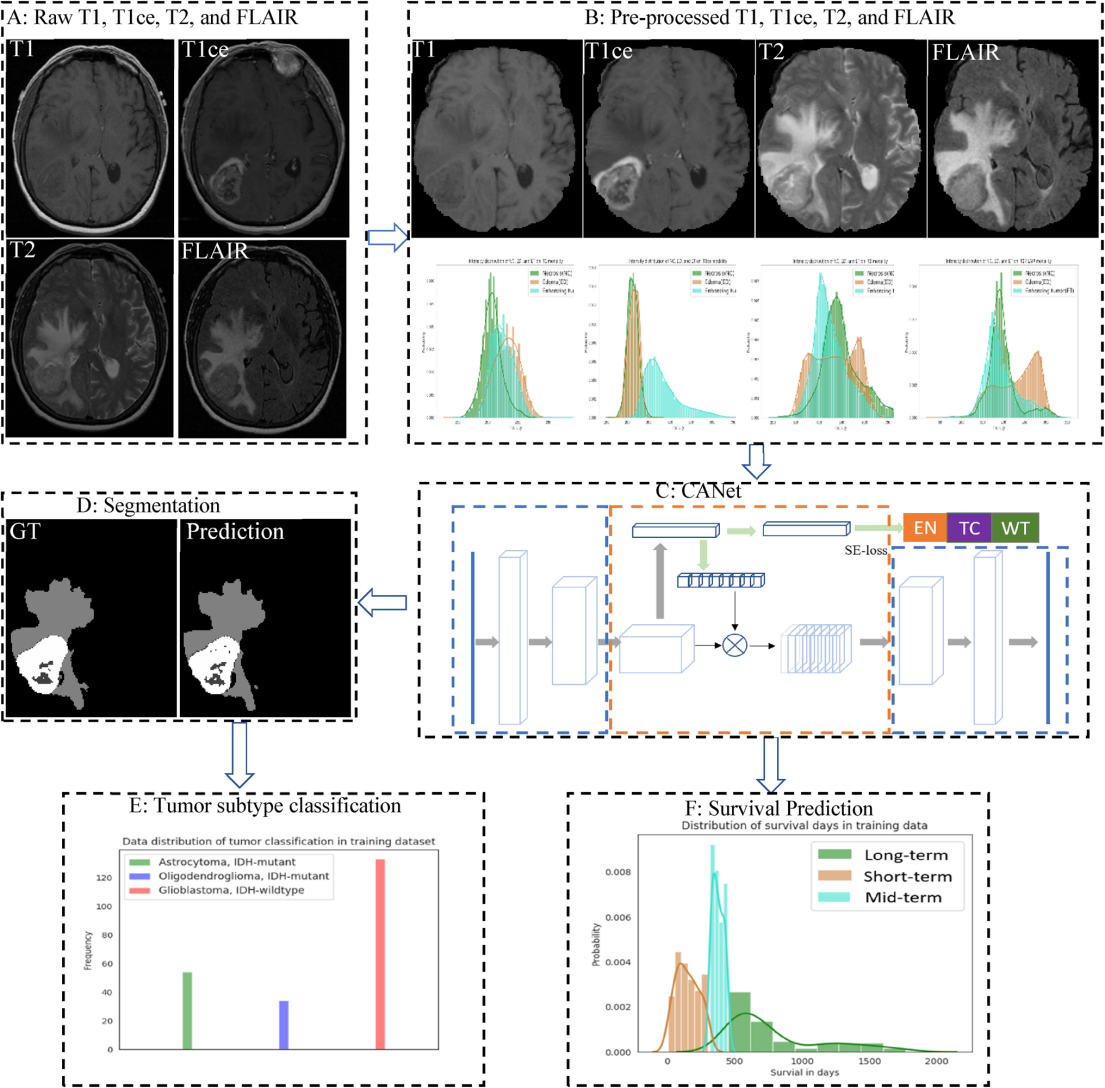
Overview of the methodology and overall work flow.

For tumor classification, the main issue is lack of data. In this work, even though we increase training sample size using data augmentation techniques, 221 cases may still be insufficient number for deep learning. Similar data shortage issue also exists in overall survival prediction. There are only 210 cases available in training phase for the CPM-RadPath 2019 Challenge.

In addition to the deep learning-based approach, we also implement overall survival prediction using a conventional machine learning method by extracting features, such as, gray-level co-occurrence matrix (GLCM), intensity, etc., then applying LASSO to select features, and finally using linear regression for survival prediction. We compare the result with that of our proposed method. The comparison shows that the proposed method achieves better performance (as shown in Table 4).

We also analyze the impact of gender and age on overall survival in this work. In the training data, patients with high-grade glioma (HGG) have 461.0314 average survival (AS) days, and 376 median survival (MS) days. Low-grade glioma (LGG) patients have 1199.8 AS with 814 MS days. We investigate impact of average age (AA), median age (MA), and gender information to average survival (AS) and median survival (MS), then compare the overall performance. The comparison results are shown in Table 5. For patients with HGG, both male and female have similar average and median age (mean age difference is less than 1 year), but male patients have much more AS days (520.6 versus 433), as well as MS days (426.5 versus 291). However, female patients with LGG (about 3 years younger) have longer AS period and MS period. Overall, regardless of tumor grade, male patients that are older have fewer survival days as shown in our experimental data.

We also conduct statistical analysis on the impact of gender and age to overall survival using analysis of variance (ANOVA). The p-value is shown in Table 6. The statistical analysis suggests that gender and age are not significant for overall survival for this dataset with only 106 patients.

## Method

There are many methods reported in literature on brain tumor segmentation that include intensity-based, atlas-based, deformable model-base, hybrid-based, and deep learning-based methods^29^. Recently, deep learning-based methods offered better performance for tumor segmentation^25,30,31^. For tumor classification, both non-invasive structural MRI and pathology images are utilized to classify brain tumors^32–34^. Overall survival prediction is to estimate the remaining life span of a patient with brain tumors. Most existing work is based on traditional machine learning and linear regression^1,35^.

Figure 5 illustrates an overview of the proposed framework. In A, there are four raw MRI modalities: T1, T1ce, T2, and FLAIR. The raw images are pre-processed in B, including co-registration, skull-stripping, noise reduction, etc. We then perform a z-score normalization for the brain region only to have zero mean and unit standard deviation. Subsequently, the proposed CANet is applied to segment tumor as shown in C. The segmentation results are shown in D. In E, a 3D CNN is utilized to classify tumor using the segmented abnormal tissues. In F, we extract high-dimensional features using front-end of CANet, and then apply a linear regression for overall survival prediction. Note that the best model of tumor segmentation may result in the best performance in tumor subtype classification and overall survival prediction. We further posit that the best model in tumor segmentation may achieve the best performance in tumor subtype classification and survival prediction, particularly since the CANet is also used as a feature extractor for these two tasks. Therefore, we proceed with the proposed CANet for tumor classification and survival prediction tasks.

**Figure 6:**
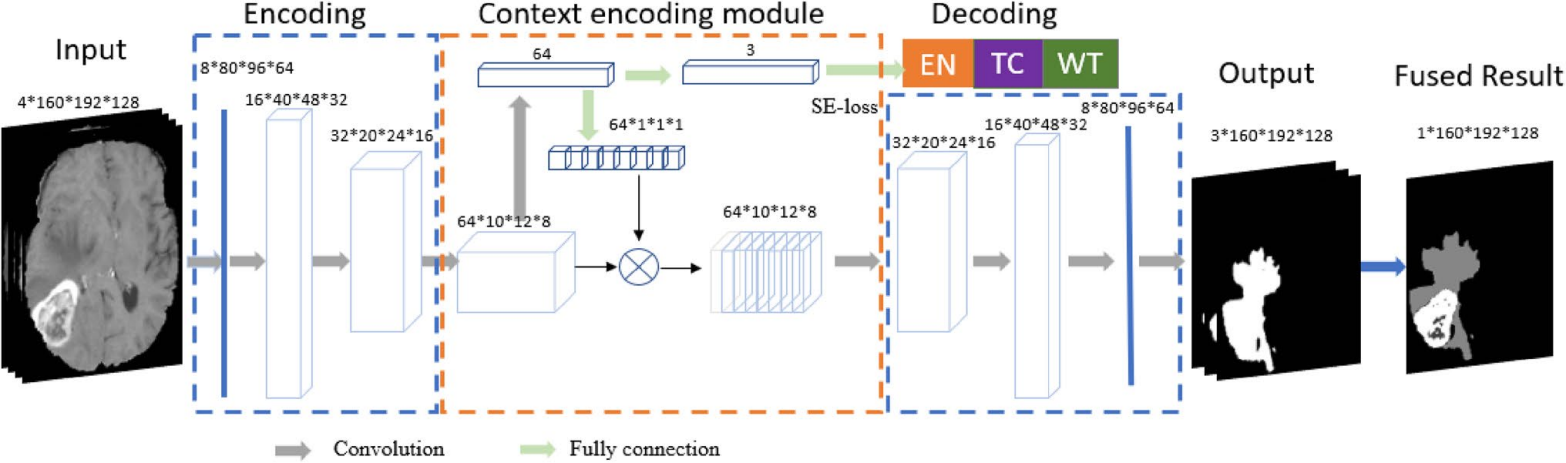
Overview of the proposed CANet architecture for tumor segmentation. It consists of encoding, decoding, and context encoding modules. Encoding and decoding module are UNet-like symmetric. Context encoding module produces semantic loss.

### Context-aware deep neural network

In this work, we introduce a Context-Aware deep neural network (CANet) architecture that integrates multiple volumetric MRI processing tasks. Inspired by the work of context encoding network^36^, the proposed architecture is substantially augmented for brain tumor segmentation^37^, tumor subtype classification, and survival prediction. The proposed CANet architecture with corresponding design parameters is illustrated in Fig. 6. A critical feature of the proposed CANet is the context encoding module, which computes scaling factors related to the representation of all classes. These factors are learned simultaneously in the training phase via the semantic loss error regularization, defined by ${L}_{se}$. The scaling factors capture global information of all classes, essentially learning to mitigate the training bias that may arise due to imbalanced class representation in data. Accordingly, the final loss function consists of 2 terms:

**Figure 7:**
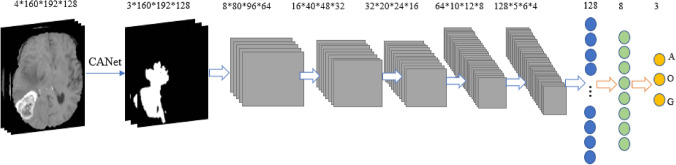
Overview of CNN-based tumor classification. The input images are first processed by CANet, and the CANet outputs (ET, WT and TC) are directly fed into the CNN-based classifier.

1 $$L={L}_{dice}+{L}_{se}$$

where ${L}_{dice}$ is a Dice calculated by the difference between prediction and ground truth, and ${L}_{se}$ is the sematic loss.

The CANet is shared among all three pipelines such as tumor segmentation, tumor subtype classification, and survival prediction, respectively, due to the inherent similarity and potential overlap of features that are useful for each task. Accordingly, encoding module of the proposed CANet is used as feature extractor for survival prediction, and the tumor subregion probability maps produced by the decoding module is used as input to the tumor subtype classification pipeline. The best model of the CANet that offers the best performance in tumor segmentation is adopted for tumor subtype classification and survival prediction pipelines, respectively.

### CNN-based tumor segmentation

An overview of the proposed context aware deep learning method for tumor segmentation is shown in Fig. 6. The proposed CANet captures global texture features and utilizes semantic loss to regularize the training error^19,36^ The architecture consists of encoding, context encoding, and decoding modules. The encoding module extracts high-dimensional features of the input. The context encoding module produces updated features and a semantic loss to regularize the model. The decoding module reconstructs the feature maps to an output prediction, so that we compute the difference between the reconstructed output and input images as a regularizer. The proposed CANet offers average DSC of 0.821, 0.895, and 0.835 for ET, WT, and TC, respectively.

### CNN-based tumor classification

The pipeline for tumor classification is shown in Fig. 7. Accordingly, the output of the CANet is directly fed into the CNN-based classifier to obtain tumor subtype classification. The classification model consists of five convolutional and pooling layers followed by two fully connected layers, and a classification layer with three outputs. All layers incorporate ReLu activation except for the classification layer, which utilizes a softmax activation function. This study considers three tumor subtypes: lower grade astrocytoma, *IDH*-mutant (A), oligodendroglioma, *IDH*-mutant, *1p/19q* codeleted (O), and glioblastoma and diffuse astrocytic glioma with molecular features of glioblastoma, *IDH*-wildtype (G). The proposed method achieves the DSC of 0.639 in testing phase. Moreover, our testing result ranked the second place in the CPM-RadPath challenge using the proposed method.

**Figure 8:**
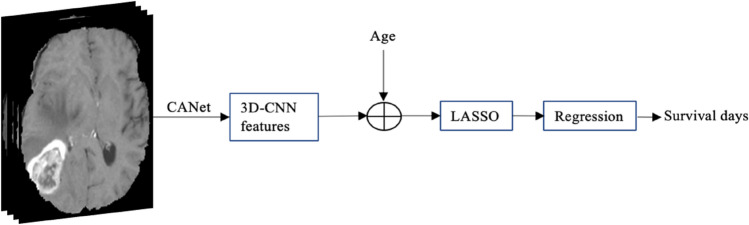
Pipeline of proposed method for overall survival prediction. The encoding module of the CANet is used to extract high dimensional features, named 3D-CNN features. We subsequently incorporate subject age as an additional feature followed by feature selection using LASSO. Finally, we estimate survival days using linear regression.

### Hybrid method for survival prediction

Instead of extracting features and using a traditional machine learning approach, we utilize the proposed CANet to extract high-dimensional features. We believe that the extracted features from tumor segmentation are associated with overall survival. We use age of the patient as an additional feature along with extracted features of the CANet. LASSO method^37^ is used for selecting more relevant features for determining the survival days of the patients. Finally, we apply a linear regression to the selected features for overall survival prediction (as shown in Fig. 8). The proposed method shows a promising result with accuracy of 0.484 in the testing phase.

### Data description

In this work, the primary experimental data is obtained from Multimodal Brain Tumor Segmentation Challenge 2019 (BraTS 2019)^1,38–41^ for brain tumor segmentation and overall survival prediction, and Computational Precision Medicine: Radiology-Pathology Challenge on Brain Tumor Classification 2019 (CPM-RadPath 2019)^42^ for tumor classification. The BraTS 2019 segmentation dataset includes training, validation, and testing data. The training data has a total of 335 cases consisting of 259 HGG and 76 LGG cases. There are 102 cases obtained from The Cancer Imaging Archive (TCIA)^43^, and rest are from a private dataset. Only cases with gross total resection (GTR) are evaluated for overall survival prediction. BraTS 2019 challenge additionally offers 125 and 166 cases for the validation and testing phases, respectively. Note that the grading information, resection status, and ground truth are privately owned by the challenge organizer and not available for public use.

In addition to BraTS 2019 testing data, we obtain 86 new patient cases from TCIA and newly released BraTS 2020 datasets to expand the overall testing dataset to 252 cases for tumor segmentation. The CPM-RadPath 2019 tumor subtype classification challenge offers 221, 35, and 73 cases training, validation, and testing, respectively. We obtain 69 new cases sourced from the BraTS 2019 segmentation dataset to scale up the overall tumor subtype classification testing dataset to 142 cases. Similarly, the BraTS 2019 survival prediction challenge offers 210, 29, 107, cases for training, validation, and testing, respectively. We include an additional 17 cases collected from the BraTS 2020 dataset to expand the survival prediction testing set to 124 cases. Note that we have obtained the maximum possible patient cases from multiple sources for each task ensuring zero redundancy. The additional testing data is used to demonstrate the generalizability of each proposed method beyond the dataset used in a specific challenge.

All ground truths for this study are established and verified by clinical experts, and the ground truths are available for only the training data. In both datasets, the multimodal MRIs have been pre-processed by the organizers following the protocol in^39^. Each patient case consists of four different MR image modalities (T1, T1ce, T2, and T2-FLAIR). Each volume is of size $240\times 240\times 155$, where 155 represents the number of slices in the volume. Moreover, the summary of gender information for both training datasets volumes is also shown in Table 7.

For segmentation, the tumor ground truth consists of one/more abnormal tissue(s): necrosis (NC), peritumoral edema (ED), and enhancing tumor (ET). However, performance evaluation is based on tumor sub-regions: enhancing tumor (ET), tumor core (TC), and whole tumor (WT), where TC consists of ET and NC. WT is a combination of TC and ED. For tumor classification, there are three subtypes: lower grade astrocytoma with *IDH*-mutant (Grade II or III), oligodendroglioma with *IDH*-mutant, *1p/19q codeleted* (Grade II or III), and glioblastoma and diffuse astrocytic glioma with molecular features of globlastoma, *IDH*-wildtype (Grade IV). For overall survival prediction, there are three categories: short-term (< 10 months), mid-term (between 10–15 months), and long-term (> 15 months).

### Experimental setup

All experiments in this study are performed in accordance with relevant guidelines and regulations as approved by the institutional IRB committee at Old Dominion University.

In the training phase of all three tasks, we apply data augmentation by randomly applying rotation (${90}^{^\circ }, {180}^{^\circ }, {270}^{^\circ }$) and scaling (factor within 0.9–1.1). Due the limitation of GPU capacity, we center crop of the resulting image to size $160\times 192\times 128$, while ensuring that the image still captures all tumor information. The final input data for the CANet is a 4-D image of size $4\times 160\times 192\times 128$ for tumor segmentation.

Experiment 1: Brain tumor segmentation. A total of 335 patients are used for training, and 125 patients are used for validation. Note that the ground truths of the validation dataset are not available to public. At the validation and testing phases, we submit the segmentation results to the challenge portal for BraTS 2019 Online evaluation^44^. For the hyperparameters of the proposed context aware deep learning, the initial learning rate is set to 0.0001, and decays gradually to 0 at the end of training. Total number of epochs is set to 300. The Adam optimizer is used^45^ for gradient descent optimization. In order to prevent overfitting in the training phase, we apply the Leaky-Relu activation function and drop out with a ratio of 0.2.

Experiment 2: Brain tumor classification. There are 221 cases provided in the training phase. We randomly take 80% of the data as training, and use the remaining 20% as our own validation set, while maintaining the same proportion of each tumor subtype in each set. The ground truth of the validation and testing data are privately held by the challenge organizer. In validation phase, we submit the results for CPM-RadPath online evaluation^46^. The hyperparameters are similar to those used in tumor segmentation, but with total number of epochs is set to 2000. Note that for the testing phase, challenge participants are required to submit the wrapped algorithm using Docker^27^, a platform to develop, deploy, and run applications inside containers, and tested by the organizer. The ranking is based on the performance evaluated by the organizer. Throughout the process, only the challenge organizer is involved in the testing evaluation.

Experiment 3: Overall survival prediction. For the training phase, we randomly split the training data into 80% and 20% sets for training and validation, respectively, while maintaining the same proportion of cases from each risk category in each set. We then apply the trained model to the validation data for online evaluation, and finally apply to the testing data for ranking. The training hyperparameters are similar to that of tumor segmentation, but with total number of epochs is set to 1000.

### Evaluation metrics

For tumor segmentation, Dice similarity coefficient (DSC) and Hausdorff distance are used to measure the segmentation quality^47^. DSC quantifies the overlap between two subsets. It is computed as $DSC=\frac{2\left|A\cap B\right|}{\left|A\cup B\right|}$^47^, where A and B are two subsets. DSC of 0 means no overlap at all between the subset A and B. DSC of 1 indicates that the subsets are perfectly overlapped. Hausdorff distance measures how far two subsets of a metric space are from each other. It is calculated as ${d}_{H}\left(A,B\right)=\mathrm{max}\left\{h\left(A,B\right), h(B,A)\right\}$, where $h\left(A,B\right)=\underset{{a\epsilon A}}{\mathrm{max}}\underset{b\in B}{\mathrm{min}}\Vert a-b\Vert$, $\Vert \bullet \Vert$ is the norm operator^26^. Smaller Hausdorff distance means that the two subsets are closer. For evaluation of tumor classification and overall survival prediction, accuracy and mean square error (MSE) are used.
